# Function and postnatal changes of dural afferent fibers expressing TRPM8 channels

**DOI:** 10.1186/s12990-015-0043-0

**Published:** 2015-06-26

**Authors:** Lynn Ren, Ajay Dhaka, Yu-Qing Cao

**Affiliations:** Washington University Pain Center and Department of Anesthesiology, Washington University School of Medicine, St. Louis, MO 63110 USA; Department of Biological Structure, Neurobiology and Behavior Graduate Program, University of Washington, Seattle, WA 98195 USA

**Keywords:** Migraine, Headache, TRPM8, CGRP, Dural afferent fibers

## Abstract

**Background:**

Genome-wide association studies have identified *TRPM8* (transient receptor potential melastatin 8) as one of the susceptibility genes for common migraine. Here, we investigated the postnatal changes of TRPM8-expressing dural afferent fibers as well as the function of dural TRPM8 channels in mice.

**Results:**

First, we quantified the density and the number of axonal branches of TRPM8-expressing fibers in the dura of mice expressing farnesylated enhanced green fluorescent protein (EGFPf) from one *TRPM8* allele between postnatal day 2 (P2) to adulthood. The number of axonal branches on individual dural EGFP-positive fibers was decreased by 30% between P2 and P11. The density of dural EGFP-positive fibers was subsequently reduced by 50% between P16 and P21. Conversely, the density and the number of branches of axons expressing calcitonin gene-related peptide remained stable in postnatal mouse dura. The density of TRPM8-expressing fibers innervating the mouse cornea epithelium was significantly increased from P2 to adulthood. Next, we tested the function of dural TRPM8 channels in adult mice and found that TRPM8 agonist menthol effectively inhibited the nocifensive behavior evoked by dural application of inflammatory mediators.

**Conclusions:**

Our results indicate that the TRPM8-expressing dural afferent fibers undergo cell- and target tissue-specific axonal pruning during postnatal development. Activation of dural TRPM8 channels decreases meningeal irritation-evoked nocifensive behavior in adult mice. This provides a framework to further explore the role of postnatal changes of TRPM8-expressing dural afferents in the pathophysiology of pediatric and adult migraine.

**Electronic supplementary material:**

The online version of this article (doi:10.1186/s12990-015-0043-0) contains supplementary material, which is available to authorized users.

## Background

Migraine is a common neurovascular disorder that affects more than 10% of the general population [1]. It is characterized by recurrent attacks of debilitating headaches and other neurological symptoms [2]. It is well established that the activation and sensitization of the primary afferent neurons (PANs) innervating the dura and cerebral blood vessels underlie the pathogenesis of headache [3–5]. Migraine has a strong genetic component. Recent genome-wide association studies of common migraine have discovered several susceptibility genes, including the gene encoding the transient receptor potential melastatin 8 (TRPM8) channel [6–8]. The TRPM8 single nucleotide polymorphism variant is 950 bp upstream of the transcription start site for TRPM8 mRNA [6], and has been verified in several migraine cohorts [6–8]. Whether and how it affects the expression of TRPM8 channels as well as the activity of TRPM8-expressing dural afferents is not clear.

TRPM8 belongs to the large family of transient receptor potential non-selective cation channels and is activated by chemical cooling agents and temperatures below 26°C [9]. TRPM8 channels are present in a distinct population of small-diameter PANs that do not bind to isolectin B4 and express little neuropeptide calcitonin gene-related peptide (CGRP) [10–13]. PANs expressing TRPM8 channels innervate both the skin and visceral organs [11, 14–16], and are required for the detection of cool to noxious cold temperatures as well as the expression of injury-induced cold allodynia and cooling-induced analgesia [10, 17–23]. Cold is a well-known trigger of migraine [24], and cold allodynia has been reported in migraine patients [25]. Conversely, topical application of TRPM8 agonist menthol offers pain relief in some migraine patients [26]. In rats, activation of meningeal TRPM8 channels causes cutaneous facial and hindpaw allodynia [27]. These studies implicate a potential role of cutaneous and dural TRPM8 channels in migraine pathogenesis. However, it is not clear whether TRPM8-expressing dural afferent fibers may also exert anti-nociceptive function in the setting of meningeal irritation, which may occur during episodes of migraine headache [3–5].

In a previous study, we used retrograde tracer DiI to label dural afferent neurons in adult mice expressing farnesylated enhanced green fluorescent protein (EGFPf) from one of the TRPM8 loci (*TRPM8*^*EGFPf/*+^) and found few, if any, DiI-labeled EGFP-positive dural afferent neurons [28]. Another study using the same strain of mice reported sparse innervation of the TRPM8-expressing fibers in some areas of the dura [29]. This was surprising, given the implication of TRPM8 in migraine pathophysiology by genetic and functional studies. This prompted us to quantitatively analyze the dural afferent fibers expressing TRPM8 channels to see whether they differ significantly from fibers expressing CGRP, which has a well-established role in migraine pathophysiology [30]. And if this is the case, whether the TRPM8- and CGRP-expressing dural afferents differ in neonatal mouse dura or whether they undergo differential postnatal changes. Does the activation of dural TRPM8-expressing fibers inhibit or exacerbate meningeal irritation-induced nocifensive behavior in adult mice?

In this study, we found that both the density and the number of branches of TRPM8-expressing dural afferent fibers was decreased substantially from postnatal day 2 (P2) to adulthood. The reduction occurred before the onset of puberty and was independent of the expression and/or the activation of TRPM8 channels *per se*. Conversely, neither the density nor the number of branches of CGRP-expressing fibers was altered in mouse dura from P2 to adulthood. The density of TRPM8-expressing fibers innervating the mouse cornea epithelium was significantly increased from P2 to adulthood. Our results suggest that TRPM8-expressing dural afferent fibers undergo unique cell- and target tissue-specific axonal pruning during postnatal development. Furthermore, we observed that dural application of TRPM8 agonist menthol in adult mice effectively reduced head-directed nocifensive behavior induced by dural application of inflammatory mediators (IM). Taken together, this provides a foundation for exploring the contribution of postnatal changes of TRPM8-expressing dural afferents to the pathophysiology of pediatric and adult migraine.

## Results

### The density of TRPM8-expressing fibers was significantly reduced in mouse dura between P16 and P21

**Figure 1:**
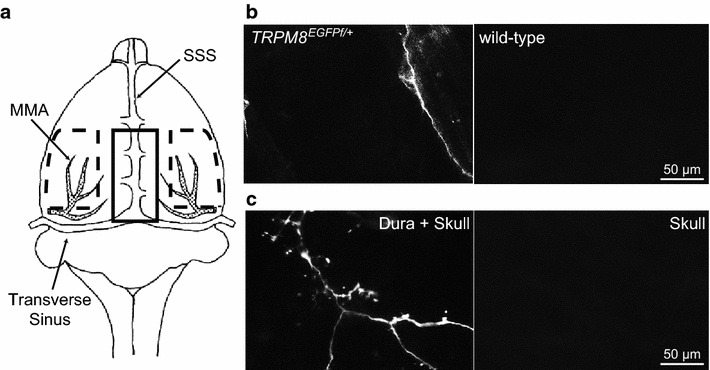
Schematic of the mouse supratentorial dura mater. **a** The midline and lateral regions were depicted as the regions within the *solid* and the *dashed lines*, respectively. *SSS* superior sagittal sinus, *MMA* middle meningeal artery. **b** *Left* EGFP-ir in the dura of an adult *TRPM8* ^*EGFPf/*+^ mouse. *Right* The absence of EGFP-ir in the dura of an adult wild-type mouse validates the specificity of the antibody. **c** *Left* EGFP signal in the dura of a P2 *TRPM8* ^*EGFPf/*+^ mouse imaged with the skull attached. *Right* no EGFP signal from the P2 *TRPM8* ^*EGFPf/*+^ mouse skull when the dura was removed.

The EGFP signal in heterozygous *TRPM8*^*EGFPf/*+^ mice corresponds well with the endogenous TRPM8 expression [11]. To fully visualize the TRPM8-expressing primary afferent axonal terminals, we stained the dura of *TRPM8*^*EGFPf/*+^ mice at various ages with the anti-EGFP antibody and quantified the density of fibers containing the EGFP immunoreactivity (EGFP-ir). Previous studies have shown a regional difference in the density of CGRP-expressing fibers innervating the dura and the cerebral vessels in rats [31, 32]. This prompted us to segregate the dura into midline and lateral regions (Figure 1a). The former contains the dura above the superior sagittal sinus (SSS) between bregma and lambda; the lateral regions include the dura covering the middle meningeal artery. For each mouse, images from 40 non-overlapping dural areas (0.15 mm^2^ each) were randomly taken for analysis: 20 in the midline region and 10 in each of the lateral region. Consistent with a previous report [29], we found EGFP-positive fibers in the dura of adult *TRPM8*^*EGFPf/*+^ mouse (Figure 1b, left). No EGFP-ir was found in the dura of adult wild-type mice, validating the specificity of the antibody (Figure 1b, right). To preserve tissue integrity, we imaged the P2 dura with the skull attached (Figure 1c, left). There was no EGFP signal left when the dura was removed from the skull of a P2 *TRPM8*^*EGFPf/*+^ mouse (Figure 1c, right), indicating that the EGFP-ir in the P2 samples originated from TRPM8-expressing axons in the dura, as opposed to from the skull.

**Figure 2:**
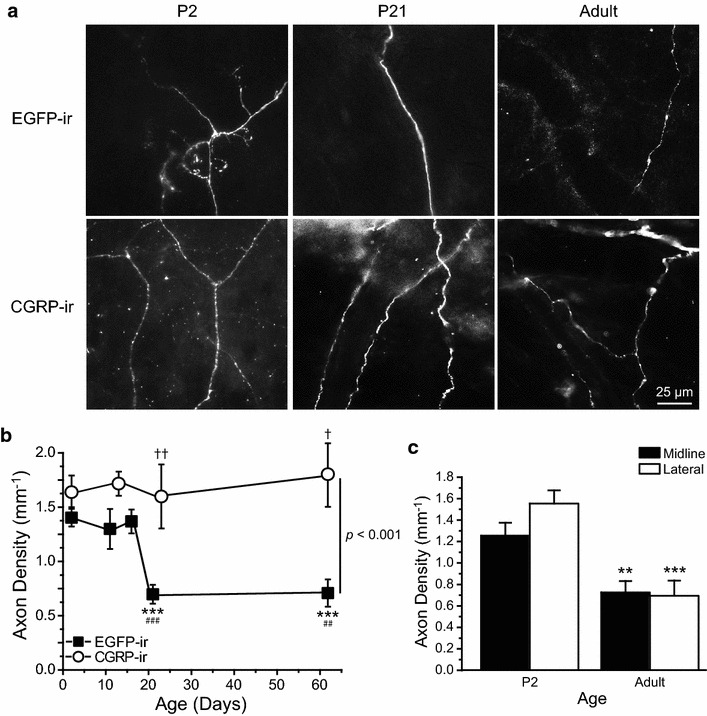
Postnatal reduction of the EGFP-positive fiber density in the dura of *TRPM8* ^*EGFPf/*+^ mice. **a** Representative images of axons containing EGFP-ir in *TRPM8* ^*EGFPf/*+^ dura and CGRP-ir in wild-type dura from P2, P21 and adult mice. **b** Average EGFP-expressing and CGRP-expressing axon densities in the dura mater of *TRPM8* ^*EGFPf/*+^ (n = 6–10 mice in each group) and wild-type mice (n = 4–10 mice in each group) between P2 and adulthood, respectively. Within the EGFP groups: one-way ANOVA overall significance *p* < 0.001, ***p < 0.001, post hoc Bonferroni test compared with the P2 group; ^##^ *p* < 0.01, ^###^ *p* < 0.001, post hoc Bonferroni test compared with the P16 group. Between EGFP and CGRP groups: two-way ANOVA overall significance *p* < 0.001, ^†^ *p* < 0.05, ^††^ *p* < 0.01, post hoc Bonferroni test between the corresponding EGFP and CGRP groups. **c** EGFP-positive axon densities in midline and lateral regions of dura mater of P2 and adult *TRPM8* ^*EGFPf/*+^ mice (n = 10 and 6 mice, respectively). **p < 0.01, ***p < 0.001, two-way ANOVA with post hoc Bonferroni test, compared with the corresponding P2 groups.

First, we compared the density of dural EGFP-positive fibers in P2 and adult *TRPM8*^*EGFPf/*+^ mice (Figure 2a). Axon density (mm^−1^) was quantified as total axon length divided by the total area sampled in each mouse. Relative to the P2 dura, the density of EGFP-positive fibers was reduced by 50% in the adult dura (Figure 2b, *p* < 0.001, one-way ANOVA with post hoc Bonferroni test). Next, a closer examination of the time course showed that the axon density remained stable from P2 to P16. The decrease of fiber density mainly occurred between P16 and P21 (Figure 2b, *p* < 0.001). Both male and female mice were included in the study, as we found no sex difference at all ages. Specifically, the density of EGFP-positive dural fibers was comparable in adult male and female mice (0.75 ± 0.20 and 0.67 ± 0.14 mm^−1^, respectively, n = 3 in each group). The lack of sex difference is not entirely unexpected, as the reduction of EGFP-positive dural fiber density occurs before the onset of puberty in mice (around P25, [33]).

Do other dural afferent fibers undergo similar postnatal changes? The meninges and cerebral arteries are densely innervated by primary afferent fibers that express neuropeptide CGRP, which plays an important role in migraine pathophysiology [30]. Previous studies have shown that TRPM8 and CGRP are expressed in different and non-overlapping subpopulations of PANs in trigeminal ganglion (TG) and dorsal root ganglion (DRG) [10–13]. Here, we quantified dural fibers that exhibited CGRP-immunoreactivity (CGRP-ir) in wild-type mice. The densities of axons containing EGFP-ir and CGRP-ir were similar in P2 dura (Figure 2b). Unlike TRPM8-expressing fibers, the density of CGRP-expressing dural fibers remained stable from P2 to adulthood (Figure 2b). Consequently, the density of EGFP-positive fibers was significantly lower than that of CGRP-expressing fibers in P21 and adult mouse dura (Figure 2b, *p* < 0.01 and *p* < 0.05, respectively; two-way ANOVA with post hoc Bonferroni test). These data indicate that the decrease of fiber density is a unique feature of the TRPM8-expressing dural afferent neurons.

Does this occur in all regions of the dura? We found that the density of EGFP-positive axons in the midline and lateral regions were comparable in both P2 and adult *TRPM8*^*EGFPf/*+^ mice (Figure 2c). From P2 to adulthood, dura mater in both regions exhibited a 50% reduction of the EGFP-positive fiber density (Figure 2c, *p* < 0.001, two-way ANOVA on the effect of age). On the contrary, the density of CGRP-expressing axons was comparable between P2 and adult dura in both the midline and lateral regions (data not shown).

### The axonal branching of TRPM8-expressing dural fibers was decreased in adult mice

**Figure 3:**
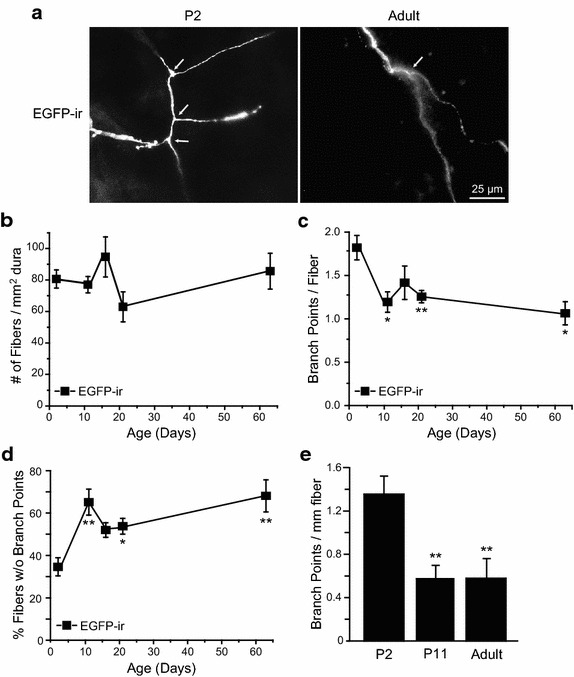
Postnatal reduction of the axonal branching of EGFP-positive fibers in the dura of *TRPM8* ^*EGFPf/*+^ mice. **a** Representative images of axons containing EGFP-ir in the dura of P2 and adult *TRPM8* ^*EGFPf/*+^ mice. Each image contains one fiber. *Arrows* indicate individual branch points on the fiber. **b** The average number of EGFP-positive fibers per mm^2^ of mouse dura (n = 5–10 mice in each group, p = 0.17, one-way ANOVA). **c** The average number of branch points on individual dural EGFP-positive fibers between P2 and adulthood (same mice as in **b**). *p < 0.05, **p < 0.01, one-way ANOVA with post hoc Bonferroni test, all compared with the P2 group. **d** The percentage of dural EGFP-positive fibers without branch points between P2 and adulthood (same mice as in **b**). *p < 0.05, **p < 0.01, one-way ANOVA with post hoc Bonferroni test, all compared with the P2 group. **e** The number of branch points per mm of EGFP-positive fibers in P2, P11and adult mouse dura (n = 6–10 mice in each group). **p < 0.01, one-way ANOVA with post hoc Bonferroni test.

The reduction of EGFP-positive dural fiber density in postnatal *TRPM8*^*EGFPf/*+^ mice may result from a decrease of the number of fibers innervating the dura and/or a decrease of the length of individual fibers. We found that the number of EGFP-positive fibers per mm^2^ dura was stable from P2 to adulthood (Figure 3b, p = 0.17, one-way ANOVA). One caveat is that, since we took random images of the dura, it is possible that distant branches of the same TRPM8-expressing fiber might be counted as individual fibers.

We proceeded to quantify the branching pattern of EGFP-positive fibers in P2 and adult mouse dura. Since we did not follow individual fibers from the point they entered the dura mater, we were not able to determine the order of branches. At each branch point, the EGFP-positive fibers always bifurcated, never dividing into more than two branches (Figure 3a). Thus, the number of branch points on individual fibers corresponded to the total number of axon branches. From P2 to adulthood, the number of branch points on individual EGFP-positive fibers was decreased by 30% (Figure 3c, *p* < 0.05, one-way ANOVA with post hoc Bonferroni test between P2 and adult EGFP groups). This mainly occurred between P2 and P11 (Figure 3c, *p* < 0.05, between P2 and P11 groups), prior to the reduction of fiber density (Figure 2b). The percentage of EGFP-positive fibers without branches nearly doubled from P2 to P11 (Figure 3d, *p* < 0.01, one-way ANOVA with post hoc Bonferroni test) and remained elevated through adulthood (Figure 3d, *p* < 0.01, between P2 and adult groups), suggesting that the decrease of axon branching is not a secondary consequence of reduced axon length. To further test this hypothesis, we normalized the number of branch points for axon length and found it was still significantly decreased in P11 and adult mouse dura relative to the P2 samples (Figure 3e, *p* < 0.01, one-way ANOVA with post hoc Bonferroni test). Taken together, these data suggest that the decrease of TRPM8-expressing fiber density in adult mouse dura likely results from the reduction of terminal axon branching and, consequently, the reduction in the length of individual fibers.

**Figure 4:**
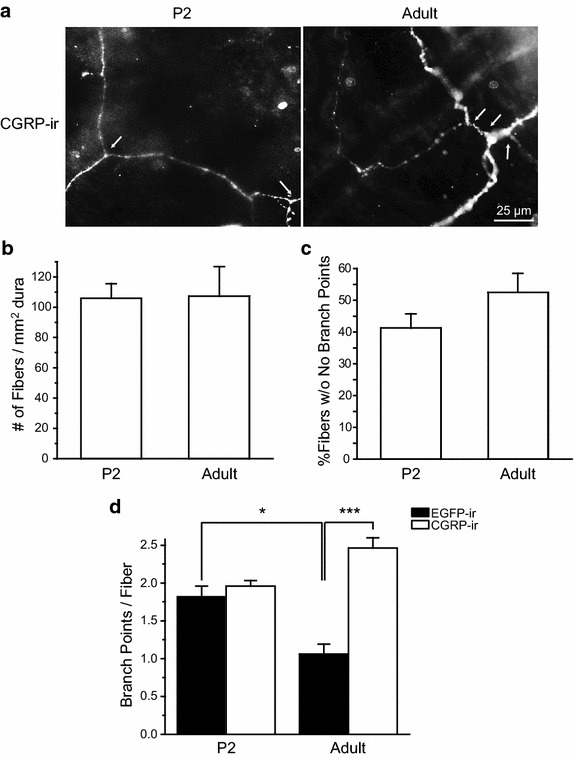
The axonal branching of CGRP-positive fibers is stable in P2 and adult mouse dura. **a** Representative images of axons containing CGRP-ir in the dura of P2 and adult wild-type mice. Each image contains one fiber. *Arrows* indicate individual branch points on the fiber. **b** The average number of CGRP-positive fibers per mm^2^ of P2 and adult mouse dura (n = 10 and 6 mice, respectively). **c** The percentage of CGRP-positive fibers without branch points in P2 and adult mouse dura (same mice as in **b**, *p* = 0.41, two-tailed *t*-test). **d** The average number of branch points per EGFP- or CGRP-positive fiber in P2 and adult mouse dura (same mice as in Figures 3e and 4b). *p < 0.05, ***p < 0.001, two-way ANOVA with post hoc Bonferroni test.

Do CGRP-expressing dural afferent fibers undergo similar changes in axon branching? The number of CGRP-positive fibers per mm^2^ dura was similar in P2 and adult mice (Figure 4b). Like the EGFP-positive fibers, the CGRP-positive fibers also bifurcated at the branch points (Figure 4a). In P2 dura, the number of branch points on individual CGRP-expressing fibers was comparable to that on EGFP-positive fibers (Figure 4d). In adult dura, individual CGRP-expressing fibers contained significantly more branch points than EGFP-positive fibers (Figure 4d, *p* < 0.001, two-way ANOVA with post hoc Bonferroni test). From P2 to adulthood, the number of branch points on CGRP-positive fibers did not increase significantly (Figure 4d, *p* = 0.070). The percentage of CGRP-positive fibers without branches did not change either (Figure 4c, *p* = 0.41, two-tailed *t* test), indicating that the decrease of axon branching is unique of the TRPM8-expressing dural afferent fibers.

### The postnatal change of TRPM8-expressing dural afferent fibers was independent of TRPM8 channel expression and/or activation

**Figure 5:**
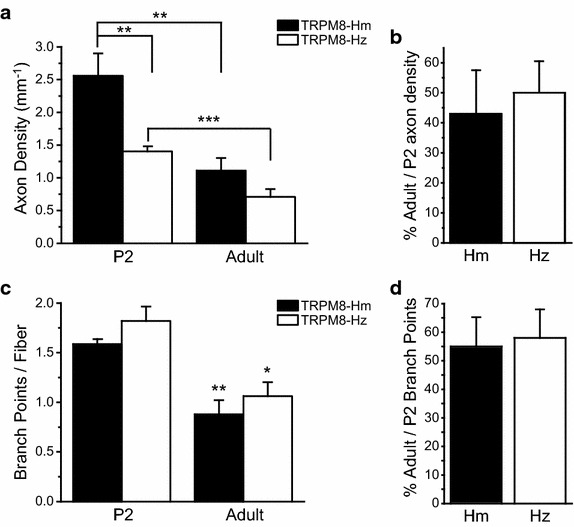
The postnatal change of EGFP-positive dural afferent fibers in *TRPM8* ^*EGFPf/*+^ and *TRPM8* ^*EGFPf/EGFPf*^ mice. **a** EGFP-positive fiber densities in the dura of P2 and adult *TRPM8* ^*EGFPf/*+^ (TRPM8-Hz, same data as in 2B EGFP groups) and *TRPM8* ^*EGFPf/EGFPf*^ mice (TRPM8-Hm, n = 8 and 6 mice in P2 and adult groups, respectively). **p < 0.01, ***p < 0.001, two-way ANOVA with post hoc Bonferroni test. **b** Percentage of adult versus P2 EGFP-positive axon densities in TRPM8-Hz and TRPM8-Hm mice (same mice as in **a**). **c** The average number of branch points per EGFP-positive fiber in the dura of P2 and adult TRPM8-Hm (same mice as in **a**) and TRPM8-Hz mice (same data as in Figure 4d EGFP groups). *p < 0.05, **p < 0.01, two-way ANOVA with post hoc Bonferroni test, compared with the corresponding P2 groups. There is no difference between TRPM8-Hz and TRPM8-Hm groups at P2 (*p* = 0.53) or adulthood (*p* = 1.5). **d** Percentage of adult versus P2 branch points per EGFP-positive fiber in TRPM8-Hz and TRPM8-Hm mice (same mice as in **c**).

Here, we compared the postnatal changes of EGFP-positive dural afferent fibers in heterozygous *TRPM8*^*EGFPf/*+^ and homozygous (*TRPM8*^*EGFPf/EGFPf*^) mice. *TRPM8*^*EGFPf/*+^ mice express TRPM8 channels from one allele and EGFPf proteins from the other. The EGFP-positive DRG neurons respond to both cold and menthol [11]. *TRPM8*^*EGFPf/EGFPf*^ mice do not express endogenous TRPM8 proteins and, instead, express EGFPf proteins from both alleles. The EGFP-ir was stronger in the dura of *TRPM8*^*EGFPf/EGFPf*^ mice than that of *TRPM8*^*EGFPf/*+^ mice, consistent with a previous report [11]. At P2, the density of EGFP-positive fibers on the dura was 82% higher in *TRPM8*^*EGFPf/EGFPf*^ mice than in *TRPM8*^*EGFPf/*+^ (Figure 5a, *p* < 0.01, two-way ANOVA with post hoc Bonferroni test). In adult mice, the density of EGFP-positive fibers was 57% higher in *TRPM8*^*EGFPf/EGFPf*^ dura than in *TRPM8*^*EGFPf/*+^ dura but this was not statistically significant (Figure 5a, *p* = 0.36). Importantly, much like what we observed in *TRPM8*^*EGFPf/*+^ mice, the density of EGFP-positive fibers in adult *TRPM8*^*EGFPf/EGFPf*^ mice was significantly reduced to approximately 43% of that in their P2 counterparts (Figure 5b). Likewise, the number of branch points on individual EGFP-positive fibers was significantly decreased from P2 to adulthood in *TRPM8*^*EGFPf/EGFPf*^ mice (Figure 5c, *p* < 0.01, two-way ANOVA with post hoc Bonferroni test). The magnitude of reduction was comparable in *TRPM8*^*EGFPf/*+^ and *TRPM8*^*EGFPf/EGFPf*^ mice (Figure 5d). Taken together, these results suggest that the postnatal reduction of TRPM8-expressing dural afferent fiber density and axonal branching may not require the expression and/or the activation of TRPM8 channels *per se*.

### The density of TRPM8-expressing fibers was significantly increased in the basal epithelium of mouse cornea from P2 to adulthood

**Figure 6:**
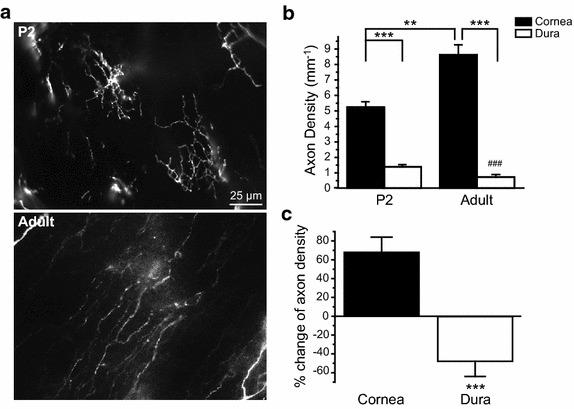
Postnatal increase in the EGFP-positive fiber density in the corneal epithelium of *TRPM8* ^*EGFPf/*+^ mice. **a** Representative images of axons containing EGFP-ir in the basal epithelium of cornea in P2 and adult *TRPM8* ^*EGFPf/*+^ mice. **b** EGFP-positive fiber densities in the corneal epithelium of P2 and adult *TRPM8* ^*EGFPf/*+^ mice (n = 7 and 5 mice, respectively). The EGFP-positive fiber densities in the dura of P2 and adult *TRPM8* ^*EGFPf/*+^ mice are also plotted (same data as in 5a). **p < 0.01, ***p < 0.001, two-way ANOVA with post hoc Bonferroni test. ^###^ *p* < 0.001, compared with the P2 dura group. **c** Percentage change of EGFP-positive axon density from P2 to adulthood in the cornea and dura of *TRPM8* ^*EGFPf/*+^ mice (same mice as in **b**). The percentage change is calculated as (adult-density − P2-density)/P2-density × 100. ***p < 0.001, two-tailed *t*-test.

Does the decrease of fiber density occur in TRPM8-expressing axons projecting to other tissues? TRPM8 channels are abundantly expressed in PANs innervating the cornea and regulate ocular surface wetness in response to temperature changes [34, 35]. Here, we compared the density of EGFP-positive fibers in the corneal epithelium of P2 and adult *TRPM8*^*EGFPf/*+^ mice. The corneal epithelium is 2–3 cells thick in P2 mice [36]. Individual EGFP-positive fibers innervate the epithelium from the stroma layer and subdivide into small branches that radially spread from the point of entry (Figure 6a). The density of EGFP-positive axons in P2 corneal epithelium was more than two-fold higher than that in P2 dura (Figure 6b, *p* < 0.001, two-way ANOVA with post hoc Bonferroni test).

During postnatal development, the thickness of the corneal epithelium increases and becomes stratified [36]. At the basal epithelium, EGFP-positive fibers run parallel to each other toward the center of the cornea (Figure 6a). Individual fibers give collaterals that ascend perpendicularly toward the superficial epithelial layer, forming clusters of highly branched terminals [34, 35]. The EGFP-positive fiber density in the basal epithelium of adult cornea was significantly higher than that of P2 corneal epithelium (Figure 6b, *p* < 0.01). Compared with adult mouse dura, the EGFP-positive fiber density was tenfold higher in the basal epithelium of adult cornea (Figure 6b, *p* < 0.001). This was likely an underestimation, as we did not take into account the axon collaterals that project to the superficial layer of the adult cornea epithelium. Nonetheless, the fiber density was increased by more than 60% in corneal epithelium from P2 to adulthood (Figure 6c, *p* < 0.001, two-tailed *t*-test), indicating that the postnatal change of TRPM8-expressing dural fiber density is target tissue-specific.

### Activation of dural TRPM8 channels inhibits meningeal irritation-induced ongoing nocifensive behavior in adult mice

**Figure 7:**
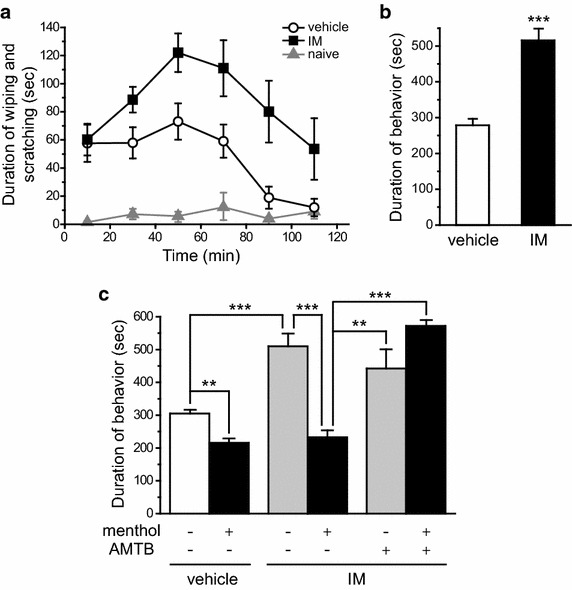
Dural application of TRPM8 agonist (-)-menthol inhibits meningeal irritation-induced ongoing nocifensive behavior in adult mice. **a** Time spent on forepaw wiping and hindpaw scratching around the scalp and periorbital area (within trigeminal V_1_ dermatome) in 20 min bins in response to dural application of vehicle or IM in adult male mice (n = 12 and 9, respectively). Naïve mice (n = 6) were habituated to the test room and recording cage as mice in other groups but were not subjected to anesthesia exposure, surgery or drug application. **b** Total duration of nocifensive behavior during the 120 min recording period in mice that received dural application of vehicle or IM (same mice as in **a**, ***p < 0.001, two-tailed *t*-test). **c** Dural application of (-)-menthol (2.8 mM in 20 µl) reduces the duration of vehicle- and IM-induced nocifensive behavior (n = 6 mice in each group; *p* < 0.001, two-way ANOVA overall effect, **p < 0.01, ***p < 0.001, post hoc Bonferroni test between individual groups). Co-application of menthol and TRPM8 antagonist AMTB (2.8 mM in 20 µl) reverses the effect of menthol (n = 3 mice; **p < 0.01, ***p < 0.001). AMTB does not alter the duration of IM-induced nocifensive behavior (*p* = 0.72, between IM and IM+ AMTB groups, n = 6 and 3 mice, respectively).

We used a behavioral assay to investigate whether and how dural TRPM8 channels regulate the gain of the migraine circuit. In rats, dural application of IM is a well-established preclinical model of headache [37–42]. First, we modified the composition of IM and applied it onto the dura of well-habituated adult male mice. The home-cage behavior of mice receiving vehicle or IM was observed for 2 h. Dural application of IM elicited robust forepaw wiping and hindpaw scratching around the scalp and periorbital area within the V_**1**_ dermatome. The duration of wiping and scratching peaked 40–60 min after IM exposure and gradually subsided (Figure 7a). Mice that received dural IM application exhibited significantly longer duration of wiping and scratching than mice treated with vehicle (Figure 7b, *p* < 0.001, two-tailed *t*-test), suggesting that meningeal irritation elicits ongoing nocifensive behavior in adult mice.

Next, we co-applied 2.8 mM TRPM8 agonist (-)-menthol along with the vehicle or IM onto the dura and recorded the duration of nocifensive behavior. Previous studies show that topical application of 1–4 mM (-)-menthol produces analgesic effects exclusively through the activation of TRPM8 channels [20, 23]. Dural application of menthol significantly reduced the duration of nocifensive behavior in both vehicle- and IM-treated mice (Figure 7c, *p* < 0 0.01 and *p* < 0.001, two-way ANOVA with post hoc Bonferroni test). It is possible that some dural afferent neurons were activated by the surgical procedure [43] and their activity was attenuated by menthol. Of note, the duration of nocifensive behavior in dural vehicle- and IM-treated groups were comparable in the presence of menthol (Figure 7c). This dose of menthol had no effect on TRPM8 knockout mice (Additional file 1: Figure S1). Dural application of TRPM8 antagonist AMTB alone did not alter the duration of IM-induced behavior (Figure 7c, *p* = 0.72). However, the effect of menthol was completely blocked by the co-application of AMTB on the dura at 1:1 molar ratio (Figure 7c), confirming that topical menthol at this concentration exerts anti-nociceptive effect through activation of TRPM8 channels. In mice receiving dural co-application of IM and WS-12, another more specific TRPM8 agonist (300 µM, [20]), the duration of nocifensive behavior was also similar to that of the vehicle group in Figure 7c (99–111% of vehicle-induced behavior, n = 4 mice).

## Discussion

In this study, we used *TRPM8*^*EGFPf/*+^ mice to investigate the postnatal changes of dural afferent fibers that express TRPM8 channels. Expression of EGFP protein corresponds well with endogenous TRPM8 expression [11]. Previous studies show that TRPM8 is predominantly expressed in a subpopulation of PANs in TG and DRG [12, 13]; only sparsely in nodose ganglion and not expressed in superior cervical ganglion neurons [44–46]. Thus, most, if not all, EGFP-positive fibers in the dura represent axons of PANs projecting from the TG.

In P2 mouse dura, both the density and the number of branches of TRPM8-expressing fibers are comparable to those of CGRP-expressing fibers, whereas they are reduced by about 50% in adult mouse dura. This is consistent with a previous report of sparse innervation of TRPM8-expressing fibers in the dura of adult *TRPM8*^*EGFPf/*+^ mice [29]. This may also account for the failure to retrogradely-label TRPM8-expressing dural afferent neurons in adult mice in our previous study [28], as sparse innervation and lack of extensive axonal branches limit the likelihood and/or the amount of tracer taken up by individual TRPM8-expressing dural afferent neurons.

Since we rely on EGFP-ir to identify TRPM8-expressing fibers, it is possible that the perceived reduction of axon density and branches is actually due to the decrease of EGFP expression that renders the EGFP-ir signal below detection threshold. This, however, is unlikely. In *TRPM8*^*EGFPf/*+^ and *TRPM8*^*EGFPf/EGFPf*^ mice, EGFP is expressed from TRPM8 loci but not fused to TRPM8 protein. Therefore, the expression of EGFP protein, but not its subcellular distribution, follows the pattern of the endogenous TRPM8 [11]. Since a differential half-life of somatic and axonal EGFP has not been reported, we assume that EGFP exhibits similar stability in soma and axon. Previous studies show that both the level of TRPM8 mRNA and the percentage of TRPM8-expressing PANs are stable in postnatal mouse PANs [46, 47]. Thus, the level of EGFP protein is likely stable in the soma as well as in the axon of postnatal mouse PANs.

In rats, there is a massive regression of the TG fiber projecting to the middle cerebral artery between P5 and P55, as the result of both cell death and axon retraction [48, 49]. However, the percentage of TRPM8-expressing PANs does not decrease postnatally [46, 47]. The number of EGFP-positive fibers per mm^2^ dura is also stable from P2 to adulthood. This argues against a significant death of the TRPM8-expressing dural afferent neurons or the retraction of TRPM8-expressing fibers in mice. Conversely, the reduction of axon branches occurs earlier than the decrease of fiber density, suggesting that axon pruning at least partially accounts for the decrease of TRPM8-expressing fiber density in adult mouse dura. A thorough characterization of the postnatal changes of the entire dural projection of single TRPM8-expressing fibers is necessary to test this model.

Neither the TRPM8-expressing cornea afferents nor the CGRP-expressing dural afferents undergo similar postnatal changes as the dural afferent fibers expressing TRPM8, suggesting that both the intrinsic regulators in TRPM8-expressing neurons and target tissue-derived molecules contribute to the reduction of TRPM8-expressing dural afferents. However, it is unlikely that the TRPM8 channel *per se* is involved. Whereas TRPM8 is expressed in *TRPM8*^*EGFPf/*+^ but absent in *TRPM8*^*EGFPf/EGFPf*^ mice [11], the magnitudes of fiber density and branch point reduction in these mice are comparable from P2 to adulthood. That said, it is important to verify that TRPM8-expressing dural afferents in wild-type mice exhibit similar postnatal changes, as the TRPM8 protein level in *TRPM8*^*EGFPf/*+^ neurons is 50% of that in wild-type [17] and the heterozygous mice display impaired cold behaviors [19]. Altogether, more experiments are needed to elucidate the mechanisms underlying the postnatal changes of TRPM8-expressing dural afferent fibers.

In addition to the morphological analysis of dural TRPM8-expressing fibers, we directly tested the function of dural TRPM8 channels, using IM to activate and/or sensitize the dural afferent neurons in adult mice [5]. In rats, dural application of IM is a well-established preclinical model of headache. It produces an aversive state of cephalic pain that can be unmasked in assays that measure motivated behavior to seek relief [50]. Other dural IM-induced behaviors include prolonged facial and hindpaw mechanical allodynia, a reduction of exploratory behavior, an increase in the duration of resting period as well as a brief facial grooming with hindpaw [37, 39, 41, 42]. We observed that dural application of IM in mice elicited longer duration of head-directed nocifensive behavior compared with the vehicle treatment. The duration of nocifensive behavior correlated positively with the number of neurons expressing FOS protein in the cervical/medullary dorsal horn in individual mice ([51], Huang et al. manuscript in preparation). Importantly, both IM-induced behavior and dorsal horn FOS expression was reduced to the control level by the pretreatment of anti-migraine drugs sumatriptan and the CGRP antagonist ([51], Huang et al. manuscript in preparation), suggesting that dural IM-induced nocifensive behavior in mice may correspond to the onging headache in humans.

Using this behavioral model, we report for the first time that activation of dural TRPM8 channels by menthol exerts anti-nociceptive effect and reduces IM-induced behavior to the control level. This is consistent with previous studies indicating that cutaneous TRPM8 channels mediate cooling-induced analgesia in the setting of tissue- and nerve injury-induced chronic pain [17–20, 22, 23]. Furthermore, TRPM8 has been shown to form complexes with the 5-HT 1B receptor, a target of the triptan family of anti-migraine drugs, and amplify the analgesic effects of 5-HT 1B agonists [52]. It will be of interest to test whether co-administration of TRPM8 and 5-HT 1B agonists exhibits a more profound anti-nociceptive effect compared with the single drug treatment. The migraine-associated TRPM8 single nucleotide polymorphism variant is 950 bp upstream of the transcription start site for TRPM8 mRNA [6]. Whether and how it affects the expression of TRPM8 channels as well as the activity of TRPM8-expressing dural afferents also merits further study.

Previous studies show that inflammatory agents such as bradykinin and prostaglandin E_2_ (PGE_2_) activate/sensitize TRPV1 channels but inhibit TRPM8 channel activity [22, 53, 54]. It is possible that the TRPM8 channels on the dura are inhibited by IM that contains bradykinin and PGE_2_. This is in agreement with our finding that co-application of the TRPM8 antagonist AMTB with IM does not alter IM-induced behavior. Future experiments are needed to test whether IM indeed inhibits the endogenous dural TRPM8 channels and whether this is necessary for the exhibition of IM-induced nocifensive behavior.

On the other hand, it is well established that cutaneous TRPM8-expressing fibers not only mediate cooling-induced analgesia, but also encode cold pain and injury-induced cold allodynia [10, 17–19, 21]. Similarly, activation of meningeal TRPM8 channels in rats causes cutaneous facial and hindpaw allodynia [27], suggesting that preferential activation of dural TRPM8 channels/fibers may encode headache. In addition to cold and cold temperatures, TRPM8 can also be activated by various endogenous phospholipids as well as testosterone [55–60]. It is possible that some migraine triggers may change the composition of phospholipids and/or the level of testosterone in local milieu, thereby altering the activation state of TRPM8 channels in dural afferent fibers as well as the excitability of these neurons. Further work is needed to identify the endogenous factors that activate dural TRPM8 channels.

Due to the lack of a mouse model of pediatric migraine, our study did not directly investigate the functional relevance of the reduction of TRPM8-expressing dural afferent fibers before the onset of puberty. We speculate that, in response to migraine triggers, the strength of excitatory inputs from dural CGRP-expressing fibers may be relatively stable from birth to puberty; whereas the strength of inhibitory tone provided by the dural TRPM8-expressing fibers may decrease significantly as the result of reduction of fiber density and axonal branching. The overall effect would be an age-dependent reduction of the activation threshold and/or an increase in the gain of the migraine circuit. This model needs to be tested after the establishment of a mouse model of pediatric migraine in the future. Of note, the prevalence of migraine in humans increases significantly from childhood to adulthood in both males and females [1]. More experiments are necessary to investigate whether similar postnatal changes of TRPM8-expressing fibers occur in human dura and, if so, whether a causal relationship exists between the decrease of dural TRPM8-expressing fibers and the increase in migraine prevalence; whether TRPM8 agonists are more efficacious in treating pediatric migraine.

## Conclusions

In this study, we show that dural afferent fibers that express TRPM8 channels undergo unique cell- and target tissue-specific axonal pruning during postnatal development in mice. Activation of dural TRPM8 channels effectively inhibits meningeal irritation-evoked nocifensive behavior in adult mice. This provides a foundation to further investigate the contribution of postnatal changes of TRPM8-expressing dural afferents to the pathophysiology of pediatric and adult migraine.

## Methods

### Mice

All procedures were carried out in strict accordance with the recommendations in the Guide for the Care and Use of Laboratory Animals of the National Institutes of Health and the guidelines of the Animal Study Committee at Washington University in St. Louis. Mice were housed on a 12-h light–dark cycle with food and water available ad libitum at the animal facility of Washington University in St. Louis. Wild-type, *TRPM8*^*EGFPf/*+^ and *TRPM8*^*EGFPf/EGFPf*^ mice on CD-1 background (backcrossed for seven generations) were used at various ages, from P2 to adult (9 weeks old). The genotype was determined by PCR of tail DNA [11]. Adult male CD-1 mice (8–10 weeks old) were used in the behavioral experiments.

### Tissue preparation

Adult mice were euthanized by barbiturate overdose (200 mg/kg, i.p.) and transcardially perfused with warm 0.1 M phosphate-buffered saline (PBS, pH 7.4) followed by cold 4% formaldehyde in 0.1 M phosphate buffer (pH 7.4) for fixation. The skull and the attached supratentorial dura mater were removed and post-fixed in 4% formaldehyde for 2 h at 4°C. The P11–P21 mice were euthanized by barbiturate overdose (200 mg/kg, i.p.). The skull with the supratentorial dura was immediately removed and fixed in 4% formaldehyde for 2 h at 4°C. Afterwards, the fixed dura from P11 to adult mice was carefully dissected from the skull using forceps. The P2 mice were euthanized by decapitation and the skull with the supratentorial dura was immediately removed and fixed in 4% formaldehyde at 4°C for 2 h. To maintain the integrity of the dura, we did not remove the skull from the P2 samples.

For cornea dissection, adult mice were euthanized and the eyeballs were removed from the skull. The corneas were removed from the eyeballs under a dissecting microscope and were fixed in 4% formaldehyde for 1 h at 4°C [34]. To dissect P2 cornea, the eyeballs were removed from euthanized mice and were fixed in 4% formaldehyde for 15 min at 4°C. The corneas were then carefully dissected from the eyeballs and were fixed in 4% formaldehyde for an additional hour at 4°C [36].

### Immunohistochemistry

The fixed dura and cornea samples were washed three times in 0.1 M PBS and were then incubated in blocking buffer (10% normal goat serum, 0.3% Triton X-100, 0.01 M Tris–HCl and 0.01 M PBS, pH 7.4) at room temperature. This was followed by overnight incubation in the primary antibody diluted in blocking buffer at 4°C. The samples were then washed 6 times (5 min per wash) in wash buffer (1% normal goat serum, 0.3% triton X-100, 0.01 M Tris and 0.01 M PBS, pH 7.4) at room temperature. Samples were blocked in blocking buffer for 1 h at room temperature, followed by 1 h incubation in the secondary antibody diluted in blocking buffer at room temperature. The samples were then washed six times in wash buffer and rinsed three times in 0.01 M PBS.

Dura samples from P2 mice were mounted on the slides with the skull attached. All other dura samples were carefully spread out on gelatin-coated slides using camel hair brushes. Cornea samples were cut into a flower shape and then mounted on the slides. Samples were cover-slipped using Fluoromount-G Mounting Medium (Electron Microscopy Sciences), sealed with nail topcoat, and stored at 4°C.

The primary antibodies used were rabbit anti-CGRP (Peninsula) at 1:1,000 dilution and rabbit anti-EGFP (Invitrogen) at 1:1,000 dilution. The Alexa Fluor 568-conjugated goat anti-rabbit secondary antibody (Invitrogen) was used at 1:2,000 dilution.

### Image acquisition and analysis

Dura and cornea samples were observed through a 40× objective on a Nikon TE2000S inverted epifluorescence microscope. Images were captured with the attached CoolSnapHQ^2^ camera (Photometrics). Forty non-overlapping dura images were randomly taken per mouse (Figure 1a). Twenty non-overlapping cornea images were randomly taken per mouse, 10 from each cornea. Fiber density and branch points were measured using SimplePCI software (Hamamatsu). No image manipulations were performed except for the contrast and brightness adjustments of the representative images. Image analysis was done with experimenter blinding to the genotype and age groups.

### Surgical preparation and behavioral tests

Adult male CD-1 mice (8–10 weeks old) for behavioral tests were housed in the animal facility for at least 7 days before acclimation. Mice were transported to the testing room and were habituated individually in a clean cage (with bedding, food and water ad libitum) for 3–5 days (>3 h per day) before the surgery and behavioral tests. Mice were gently handled at least five times during each habituation period until they show no signs of freezing or rapid escaping when approached by the experimenter.

The surgery procedure was adapted from our previous study using retrograde tracers to label dural afferent neurons in mice [28]. On the test day, mice were acclimated individually in a clean cage (with bedding, food and water ad libitum) for 1 h. Subsequently, mice were anesthetized with 3–4% isoflurane in an induction chamber till losing the righting reflex and were mounted on a Stoelting stereotaxic apparatus. Anesthesia was maintained by 1.5–2% isoflurane through a nose cone. Body temperature was maintained by placing mice on a 37°C circulating water warming pad. A small amount of eye drops was placed in the eyes to prevent the corneas from drying. Lidocaine hydrochloride jelly (2%) was applied on the skin for 5–10 min before a longitudinal skin incision was made to expose the cranium. A craniectomy (~2 mm diameter) was made with a surgical blade in the area overlying the SSS between bregma and lambda, leaving the underlying dura exposed but intact [61]. Topical lidocaine solution (2%) was repetitively applied on the skull during the craniectomy to prevent the activation and/or sensitization of the primary afferent neurons. A sterile polypropylene ring was sealed to the skull surrounding the exposed dura by a mixture of dental cement powder (Stoelting 51459) and superglue adhesive to prevent the spreading of the solution to other peripheral sites. The viscosity of dental cement/superglue mix kept it from spreading to the exposed dura. After waiting 5–10 min for the mix to solidify, we applied 20 µl of solutions (see below) onto the exposed dura. Subsequently, a sterile polypropylene cap was secured over the ring with bone wax to cover the exposed dura. The skin incision was closed with 5–0 silk suture. After recovery from anesthesia, mice were returned to the clean cage and their behaviors were recorded by digital video cameras for 2 h before euthanization. Digital video files were quantified off-line by the experimenter blinding to the treatments mice received. Time spent on forepaw wiping and hindpaw scratching within the mouse V_1_ dermatome (including the scalp and periorbital area) was quantified as nocifensive behavior.

### Drug application

The composition of IM was modified from previous studies [5, 40, 41]. The IM solution contained 0.5 mM capsaicin and a mixture of proinflammatory reagents including 1 mM bradykinin, 1 mM histamine, 1 mM serotonin (5-HT) and 0.1 mM PGE_2_ in artificial cerebrospinal fluid (ACSF, pH 7.2) with 2% DMSO. The ACSF consists of (in mM) 125 NaCl, 26 NaHCO_3_, 1.25 NaH_2_PO_4_, 2.5 KCl, 1 MgCl_2_, 1 CaCl_2_, and 25 glucose. The vehicle control consists of ACSF (pH 7.2) with 2% DMSO. All chemicals were purchased from Sigma, dissolved in H_2_O (bradykinin, histamine and 5-HT) or DMSO (PGE_2_ and capsaicin) at 100× concentrations and stored at −80°C in aliquots. The IM was freshly prepared from the stock solution on each day of surgery and behavioral test.

The TRPM8 agonist (-)-menthol (Sigma) and antagonist AMTB (Sigma) were dissolved in ACSF with 2% DMSO. We pretreated the dura with 20 µl of menthol (2.8 mM), AMTB (2.8 mM) or menthol plus AMTB (2.8 mM each) for 15 min. We then replaced the solution with IM/vehicle containing 2.8 mM menthol and/or 2.8 mM AMTB.

### Statistics

Data were reported as mean ± standard error of the mean. Origin 8.1 (Origin Lab) and Statistica (StatSoft) were used to perform statistical tests. Differences with *p* < 0.05 were considered statistically significant. Two-tailed Student’s *t*-test, one-way or two-way analysis of variance (ANOVA) with post hoc Bonferroni correction was used where appropriate.

## Additional file

Additional file 1:
**Figure S1.** The effects of dural IM and menthol on wild-type and TRPM8 knockout mice.

## Abbreviations

5-HT: Serotonin
ACSF: artificial cerebrospinal fluid
CGRP: calcitonin gene-related peptide
CGRP-ir: CGRP-immunoreactivity
DRG: dorsal root ganglion
EGFPf: farnesylated enhanced green fluorescent protein
EGFP-ir: EGFP immunoreactivity
IM: inflammatory mediators
P: postnatal day
PAN: primary afferent neuron
PBS: phosphate-buffered saline
PGE_2_: prostaglandin E_2_
SSS: superior sagittal sinus
TG: trigeminal ganglion
TRPM8: transient receptor potential melastatin 8
*TRPM8*^*EGFPf/*+^: heterozygous mice expressing EGFPf from one of the TRPM8 loci
*TRPM8*^*EGFPf/EGFPf*^: homozygous mice expressing EGFPf from both TRPM8 loci
